# Supplementary material for: Comparative multiomics analysis of cell physiological state after culture in a basket bioreactor
Source: Sci Rep. 2022 Nov 23;12:20161. doi: 10.1038/s41598-022-24687-4 (PMC9686226; doi:10.1038/s41598-022-24687-4)
Supplement: Supplementary file 1 — Supplementary Information 1. [file 41598_2022_24687_MOESM1_ESM.zip › raw data/Transcriptomic raw data/6.Enrichment/2.KEGG/TE1vsTC1/ALL/src/csab04610.html]

csab04610


- csab:103215685

- Down regulated genes

103215685(-1.98840718122177)

- csab:103229609

- Down regulated genes

103229609(-2.65725180480499)

- csab:103221732

- Down regulated genes

103221732(-2.06930923500636)

- csab:103231401

- Down regulated genes

103231401(-2.56891951280362)

- csab:103233571

- Down regulated genes

103233571(-1.90705123830322)

Close
